# Translational network neuroscience: Nine roadblocks and possible solutions

**DOI:** 10.1162/netn_a_00435

**Published:** 2025-03-20

**Authors:** Lucius S. Fekonja, Stephanie J. Forkel, Dogu Baran Aydogan, Pantelis Lioumis, Alberto Cacciola, Carolin Weiß Lucas, Jacques-Donald Tournier, Francesco Vergani, Petra Ritter, Robert Schenk, Boshra Shams, Melina Julia Engelhardt, Thomas Picht

**Affiliations:** Department of Neurosurgery, Charité - University Hospital, Berlin, Germany; Cluster of Excellence: “Matters of Activity. Image Space Material”, Humboldt University, Berlin, Germany; Donders Centre for Cognition, Radboud University, Thomas van Aquinostraat 4, 6525 GD Nijmegen, the Netherlands; Centre for Neuroimaging Sciences, Department of Neuroimaging, Institute of Psychiatry, Psychology and Neuroscience, King's College London, London, SE5 8AF, United Kingdom; Brain Connectivity and Behaviour Laboratory, Sorbonne Universities, Paris, 75006, France; Max Planck Institute for Psycholinguistics, Wundtlaan 4, Nijmegen, the Netherlands; A. I. Virtanen Institute for Molecular Sciences, University of Eastern Finland, Kuopio, Finland; Department of Neuroscience and Biomedical Engineering, Aalto University School of Science, Espoo, Finland; BioMag Laboratory, HUS Medical Imaging Center, University of Helsinki and Helsinki University Hospital, Helsinki, Finland; Brain Mapping Lab, Department of Biomedical, Dental Sciences and Morphological and Functional Images, University of Messina, Messina, Italy; Center for Complex Network Intelligence (CCNI), Tsinghua Laboratory of Brain and Intelligence (THBI), Tsinghua University, Beijing, China; Department of Biomedical Engineering, Tsinghua University, Beijing, China; University Hospital and Medical Faculty of the University of Cologne, Center for Neurosurgery, Cologne, Germany; Department of Perinatal Imaging and Health, School of Biomedical Engineering & Imaging Sciences, King's College London, United Kingdom; Department of Biomedical Engineering, School of Biomedical Engineering & Imaging Sciences, King's College London, United Kingdom; Department of Neurosurgery, King's College Hospital NHS Foundation Trust, Denmark Hill, London SE5 9RS, Department of Neurosurgery, King's College Hospital NHS Foundation Trust, Denmark Hill, London SE5 9RS, United Kingdom; Charité – Universitätsmedizin, corporate member of Freie Universität Berlin and Humboldt-Universität zu Berlin, Einstein Center for Neurosciences, Charitéplatz 1, 10117 Berlin, Germany; Berlin Institute of Health (BIH) at Charité - Universitätsmedizin Berlin, Charitéplatz 1, 10117, Berlin, Germany; Department of Neurology with Experimental Neurology, Charité, Universitätsmedizin Berlin, corporate member of Freie Universität Berlin and Humboldt Universität zu Berlin, Charitéplatz 1, 10117, Berlin, Germany; Bernstein Focus State Dependencies of Learning and Bernstein Center for Computational Neuroscience, 10115, Berlin, Germany; Einstein Center Digital Future, Wilhelmstraße 67, 10117, Berlin, Germany

**Keywords:** Translational medicine, Connectomics, Network neuroscience, Patients, Personalized medicine

## Abstract

Translational network neuroscience aims to integrate advanced neuroimaging and data analysis techniques into clinical practice to better understand and treat neurological disorders. Despite the promise of technologies such as functional MRI and diffusion MRI combined with network analysis tools, the field faces several challenges that hinder its swift clinical translation. We have identified nine key roadblocks that impede this process: (a) theoretical and basic science foundations; (b) network construction, data interpretation, and validation; (c) MRI access, data variability, and protocol standardization; (d) data sharing; (e) computational resources and expertise; (f) interdisciplinary collaboration; (g) industry collaboration and commercialization; (h) operational efficiency, integration, and training; and (i) ethical and legal considerations. To address these challenges, we propose several possible solution strategies. By aligning scientific goals with clinical realities and establishing a sound ethical framework, translational network neuroscience can achieve meaningful advances in personalized medicine and ultimately improve patient care. We advocate for an interdisciplinary commitment to overcoming translational hurdles in network neuroscience and integrating advanced technologies into routine clinical practice.

## INTRODUCTION

Network neuroscience is an emerging field that explores the complex architecture and functionality of interconnected brain regions (Bassett & Sporns, 2017; Rubinov & Sporns, 2010). As part of network science and a subcategory of complex networks (Noble, Curtiss, Pessoa, & Scheinost, 2023), network neuroscience aims to apply theoretical perspectives to studying multidimensional, nonlinear, and interacting neuroscientific phenomena. This often involves using data-driven models to map the complex relationships between the brain and behavior.

Brain networks can be mapped using various methods, each providing specific information about different aspects of connectivity. Structural connectivity, derived from diffusion MRI (dMRI), visualizes white matter pathways, connecting different regions of the brain. dMRI therefore offers insights into how brain regions are physically connected to process cognitive functions and behavior (Basser, Mattiello, & LeBihan, 1994; Hagmann et al., 2008; Jeurissen, Descoteaux, Mori, & Leemans, 2019; Jones, Knösche, & Turner, 2013). Functional connectivity, often measured with resting-state or task-based functional MRI (fMRI), represents statistical dependencies or correlations between brain regions, highlighting coactivation or synchronization patterns based on blood oxygenation levels (Fox & Raichle, 2007; Friston, 2011; Logothetis, 2008). Effective connectivity describes the causal influence that one brain region exerts on another, focusing on modeling dynamic causal interactions between brain areas (Friston, 2011; Valdes-Sosa, Roebroeck, Daunizeau, & Friston, 2011). Each connectivity measure has proven valuable for understanding alterations in brain networks across various disorders (Dai et al., 2019; Fornito, Zalesky, & Breakspear, 2015; Sha, Wager, Mechelli, & He, 2019; Siegel et al., 2016). For example, in stroke patients, network disruption analyses have helped predict impairments across behavioral domains, aiding in understanding lesion impact on brain function (Hope et al., 2024; Siegel et al., 2016; Talozzi et al., 2023). In Alzheimer's disease, combining structural and functional network analyses reveal how the disease affects global brain organization and function (Dai et al., 2019). This integrated perspective allows to detect subtle, widespread brain organization changes, informing potential targeted treatments (Fornito et al., 2015).

Integrating network science with neuroscience has led to the development of innovative methods for studying neural circuits, providing deeper insights into brain function related to clinical diagnoses (Barabási et al., 2023). For instance, a network analysis has shown decreased ipsilesional structural connectivity in glioma patients, offering insights into how brain tumors impact white matter pathways (Fekonja et al., 2022). Studies in schizophrenia have explored changes in resting-state complexity and hierarchical cortical networks organization, broadening the understanding of the disorder's neurobiological basis (Bassett et al., 2008; Bassett, Nelson, Mueller, Camchong, & Lim, 2012). A recent perspective on functional neuroimaging underscores the potential of integrated neuroscience to map cognitive functions within brain networks, bridging systems, cognitive, computational, and clinical neuroscience (Finn, Poldrack, & Shine, 2023). The network-based statistic approach, developed to identify differences in brain networks, enables more precise characterization of neurological and psychiatric conditions (Zalesky, Fornito, & Bullmore, 2010). This method has led to the identification of connectomic phenotypes for psychiatric disorders, bridging the gap between genetic risk factors and clinical symptoms and supporting a shift toward dimensional models of psychopathology (Fornito & Bullmore, 2012). Rather than categorical diagnoses, researchers are now using network-based models to characterize connectivity changes across psychiatric traits (Lindner et al., 2018). These network-level changes are associated with various brain disorders, showcasing network mechanisms as a core component (Stam, 2014).

Recent trends in connectomics, including machine learning applications in network neuroscience data, further advance our understanding of brain function and support improved treatment (Good et al., 2022; Petkoski, Ritter, & Jirsa, 2023; Schirner, Deco, & Ritter, 2023; Sporns & Bassett, 2018).

Emerging studies highlight the therapeutic potential of network neuroscience. For example, a study on transcranial magnetic stimulation (TMS) demonstrated that antidepressant efficacy varies with the functional connectivity strength to the subgenual cingulate, suggesting functional connectivity's role in optimizing TMS for depression (Fox, Buckner, White, Greicius, & Pascual-Leone, 2012). In epilepsy research, the network analysis of intracranial EEG data has revealed dynamic patterns associated with seizure generation, propagation, and termination. This approach provides new insights into seizure mechanisms, potentially informing presurgical planning and improving epileptogenic zones' understanding (Khambhati et al., 2015). However, despite these advances, larger scale validation studies are essential to establish these methods as clinical standards and confirm efficacy beyond current practices.

Although promising, network neuroscience faces several technical, methodological, and communication challenges that hinder rapid clinical translation (Pinto et al., 2020; Thengone, Voss, Fridman, & Schiff, 2016; H. E. Wang et al., 2023). Many challenges overlap with general neuroimaging issues, yet network-based approaches also bring unique considerations. We will examine these challenges and propose solutions. A recent review of clinical network neuroscience emphasized the need for standardization, large-scale collaborations, and clinically relevant network measures (Douw et al., 2019). Network neuroscience relies heavily on advanced neuroimaging techniques, signal modeling, connectivity estimation, and parcellation methods, introducing complexities beyond traditional neuroimaging. These include challenges in constructing meaningful brain networks (e.g., false positives or negatives), interpreting network metrics clinically, and translating network-level insights to patient-specific interventions. Variability in parcellation schemes complicates cross-study comparisons, and while measures like modularity or global efficiency offer valuable insights into network organization, their clinical applications remain limited (Bullmore & Sporns, 2009; Eickhoff, Yeo, & Genon, 2018; Fornito, Zalesky, & Bullmore, 2010; Messé, 2020). Integrating network metrics with patient-specific outcomes, such as predicting disease progression or treatment response, represents another significant hurdle (Bassett & Sporns, 2017; Fornito et al., 2015).

The goal of this work is to highlight key translational medicine barriers and propose strategies to advance progress in translational network neuroscience (TNN).

### A Systematic Approach to Translation

The journey from research to clinical practice follows distinct phases, each with unique challenges and objectives. At its foundation lies the preclinical phase (T0), where researchers investigate basic mechanisms and disease processes to identify potential therapeutic targets. Clinical validation then progresses through increasingly comprehensive trials: initial safety assessment in small groups (T1), efficacy exploration in larger cohorts (T2), and broader confirmation studies comparing outcomes to standard treatments (T3). The final phase (T4) focuses on community-wide implementation, addressing the complexities of integrating interventions across diverse healthcare settings (Blumberg, Dittel, Hafler, von Herrath, & Nestle, 2012; Fort, Herr, Shaw, Gutzman, & Starren, 2017).

TNN currently spans these early stages of this translational continuum. While promising applications in individual-level brain mapping are emerging, most approaches remain in preclinical development, requiring rigorous validation before advancing to clinical use (Gordon et al., 2017; Gratton et al., 2018; Mahmoud et al., 2024; Mulders, van Eijndhoven, Schene, Beckmann, & Tendolkar, 2015).

## ROADBLOCKS AND ENABLERS

We have identified nine critical roadblocks that currently impede the translation of network neuroscience into clinical practice (Table 1). These challenges span theoretical foundations, methodological considerations, and practical implementation issues. Each roadblock demands specific solutions to bridge the gap between research potential and clinical reality.

**Table 1.** Roadblocks and enablers summary

Roadblock	Brief explanation	Enabler
1. Theoretical and fundamental science foundations	Need for comprehensive theories to explain observations; gap between network measures and neural processes limits interpretability.	Promote interdisciplinary collaborations; fund theory development; bridge the gap between network measures and neural processes to enhance clinical interpretability.
2. Network construction, data interpretation, and validation	Challenges in constructing accurate networks due to anatomical distortions, variability, and capturing dynamic connectivity; difficulty in validating models.	Develop robust methods accounting for pathology; incorporate dynamic connectivity analyses; utilize advanced tools like BCT, GRETNA, NetworkX, DIPY, and Nipype; establish validation frameworks through multicenter studies; develop user-friendly tools and platforms for clinicians.
3. MRI access, data variability, and protocol standardization	Variability in MRI technologies and protocols leads to noncomparable data; need for high-temporal resolution data complicates acquisition; tension between standardization and leveraging advanced imaging capabilities.	Implement harmonization strategies and quantitative MRI; consider advanced imaging techniques like MEG and EEG for capturing dynamics; develop robust models that handle variability; consider locally optimized models; improve access to high-quality scanners through resource sharing and funding.
4. Data sharing	Limited capacity and integration of data systems; varying data formats; stringent data privacy regulations hinder data exchange.	Develop standardized, scalable data-sharing platforms; promote interoperability; establish privacy-compliant guidelines; use BIDS and FAIR principles; facilitate anonymized data sharing.
5. Computational resources and expertise	Insufficient computing power and storage; lack of personnel trained in software development; tools not user-friendly for clinicians; analyzing dynamic networks increases computational demands.	Upgrade IT infrastructure with scalable solutions; involve software engineers and clinicians in development; provide training programs; collaborate with academic and industry partners; optimize algorithms for efficiency; leverage cloud computing resources.
6. Interdisciplinary collaboration	Differences among disciplines hinder effective collaboration and integration into clinical workflows.	Promote interdisciplinary programs; create collaborative environments; support knowledge exchange; require interdisciplinary teams in funding opportunities.
7. Industry collaboration and commercialization	Disconnect between academic research and commercial software development; lengthy regulatory approval processes (MDR, GDPR, AI Act).	Create partnerships with medical software companies; engage with regulatory bodies; collaborate with EHR system providers; involve policymakers and patient advocacy groups; integrate TNN tools into existing clinical platforms.
8. Operational efficiency, integration, and training	Clinical workflows' focus on efficiency and cost-effectiveness creates barriers to implementing time-intensive network analyses; lack of specialized training and expertise limits adoption of advanced tools.	Implement integrated digital health records; develop adaptable analysis tools for patient-level data; incorporate AI for automation and personalization; maintain continuous feedback between researchers and clinicians; develop specific training programs; integrate network neuroscience into medical curricula; support interdisciplinary education initiatives; facilitate research alongside clinical duties.
9. Ethical and legal considerations	Complexities in obtaining informed consent; data ownership issues; ensuring patient privacy delays implementation.	Involve ethicists and legal experts; promote transparency; harmonize guidelines; develop patient-centered frameworks; ensure robust consent procedures; establish data protection policies.

### Theoretical and Fundamental Science Foundations

#### Roadblocks.

TNN currently lacks comprehensive theories to explain its multitude of observations. Without such theorization, identifying mechanisms crucial for clinical application becomes challenging. The field's reliance on abstract network measures, such as graph-theoretical measures of centrality, segregation, or integration, creates a difficulty in relating mathematical concepts to anatomical features and clinical measures. The gap between anatomical and mathematical scales, combined with the absence of frameworks that meaningfully connect network metrics to biological reality, limits the clinical interpretability of network neuroscience findings (Binder & Desai, 2011).

#### Enablers.

The development of unified theoretical frameworks offers practical solutions to bridge these conceptual gaps. Network control theory and dynamical systems modeling provide structured approaches to connect abstract network measures with neurobiological processes (Bassett & Sporns, 2017). Digital twin methodology represents a particularly promising advance, enabling precise simulation and prediction through virtual representations of physical systems (Fekonja et al., 2024). By integrating computational models with empirical data, these approaches can create mechanistic frameworks that better reflect biological reality, including crucial elements like neuroplasticity (Fekonja et al., 2024; Ritter, Schirner, McIntosh, & Jirsa, 2013; Roy et al., 2014). The field's evolution beyond descriptive strategies to predictive and control-oriented frameworks strengthens its clinical potential (Srivastava, Fotiadis, Parkes, & Bassett, 2022). Animal studies complement human neuroimaging research by providing essential validation and causal insights. Recent developments, including a comprehensive macaque connectome and high-quality chimpanzee brain imaging data, enable valuable cross-species comparisons (Eichner et al., 2024; Shen et al., 2019). These comparative approaches reveal evolutionarily preserved network features, while computational models that integrate both human and animal data are becoming increasingly important (Brynildsen, Rajan, Henderson, & Bassett, 2023; van den Heuvel, Bullmore, & Sporns, 2016). Though translating these insights to patient care remains challenging, future research emphasizing parallel experimental designs across species and improved noninvasive neuroimaging methods shows promise.

### Network Construction, Data Interpretation, and Validation

#### Roadblocks.

Network construction in clinical populations is challenging (de Reus & van den Heuvel, 2013; Fekonja et al., 2022). For instance, in patients with brain lesions, standard atlas-based parcellations often prove inadequate, requiring adaptive network node definition strategies (Fornito et al., 2015). The construction of meaningful brain networks depends critically on methodological choices, from brain atlas selection to connectivity estimation approaches, with each decision potentially affecting research outcomes. Network measures show particular sensitivity to variations in data acquisition and processing, complicating standardization efforts (Botvinik-Nezer et al., 2020; Hallquist & Hillary, 2018). The relationship between network properties and brain dysfunction remains complex and often indirect, making clinical interpretation of network measures particularly challenging. The challenge of capturing dynamic connectivity changes over time and across different cognitive states adds another layer of complexity. Traditional analyses, assuming static connectivity, fail to reflect the brain's inherent temporal fluctuations (Hutchison et al., 2013). Tool diversity in network analysis introduces additional heterogeneity in data interpretation. Perhaps most critically, current tools excel at group-level insights but lack the adaptability needed for individual patient analysis, creating a significant barrier to personalized medicine (Croxson, Forkel, Cerliani, & Thiebaut de Schotten, 2018; Forkel, Friedrich, Thiebaut de Schotten, & Howells, 2022; Gabrieli, Ghosh, & Whitfield-Gabrieli, 2015).

#### Enablers.

The development of robust analytical tools requires interdisciplinary collaboration. Engineers, computer scientists, neuroanatomists, and clinicians working together can create solutions optimized for clinical usability. Comprehensive user documentation and tutorials simplify the creation and interpretation of complex network data.^1^^,^^2^^,^^3^ Normative models support data interpretation and are particularly valuable where disease-related anatomical changes alter typical brain structures (Srivastava et al., 2022). Robust methods for accurate definition of white matter pathways and functional nodes and edges in networks, even in the presence of lesions, are necessary. Lesion filling methods and clinical data synthesis toolboxes enable better brain segmentation and parcellation results by cropping out lesions or harmonizing image quality (Iglesias et al., 2021, 2023; Matsulevits et al., 2024; Radwan et al., 2021). Dynamic functional connectivity analyses, including sliding window techniques and time-varying graph metrics, are essential for understanding brain network dynamics, though careful validation remains important (Allen et al., 2014; Basile et al., 2022).

The field benefits from a rich ecosystem of computational tools. Several computational software now facilitate brain network construction and analysis. Examples like MRtrix3 and DSI Studio offer tools for structural connectivity, while the CONN Toolbox and Nilearn specialize in functional connectivity among many others (Abraham et al., 2014; Tournier et al., 2019; Whitfield-Gabrieli & Nieto-Castanon, 2012).^4^ Brain Connectivity Toolbox, GRETNA, and NetworkX represent just a few of the many valuable resources offering complementary capabilities for complex network analysis, dMRI analysis, and workflow integration, respectively (Hagberg, Swart, & Schult, 2008; Rubinov & Sporns, 2010; J. Wang et al., 2015). These developments exemplify network neuroscience's rapidly evolving nature, with new platforms continuously emerging to address specific analytical challenges. A comprehensive list of all available tools is beyond the scope of this article.

### MRI Access, Data Variability, and Protocol Standardization

#### Roadblocks.

The implementation of network analyses in clinical settings is influenced by variation challenges in MRI infrastructure that span research and clinical environments. Advanced research centers with dedicated systems like ultrahigh field or high-gradient scanners contrast facilities with standard clinical equipment. While dMRI benefits primarily from high-gradient capabilities, functional connectivity gain advantages from higher field strengths. This dichotomy in MRI access creates disparities between research and clinical realms, with even more pronounced differences at the global level (Bates et al., 2023; Liu et al., 2021). Equipment differences, including magnetic field strength, manufacturer variations, scanner age, and coil types, result in data that resist direct comparison across research sites. The absence of standardized protocols are major challenges to advancement and hampers the creation of large, reliable datasets, often limiting studies to small, albeit homogeneous, clinical samples (Button et al., 2013). Processing and analysis pipeline variations introduce additional complexity, further challenging result integration across studies.

#### Enablers.

While complete harmonization represents an ideal goal, developing center-specific models offers a practical intermediate solution. Hospital-specific predictive models using local neuroimaging protocols and patient populations can achieve high accuracy and immediate applicability. The challenge lies in balancing such locally optimized solutions with the need for generalizable knowledge.

Methods robust to acquisition and processing variability show particular promise. Dataset stacking and transfer learning approaches have demonstrated success in network neuroscience, with studies showing that deep learning models trained on multisite fMRI data can generalize effectively across different protocols and populations (He et al., 2020).

Harmonization strategies remain crucial for standardizing data from different acquisition protocols. Progress in dMRI has yielded robust data harmonization methods (Pinto et al., 2020). Minimum common protocols for each modality, adjustable for site and scanner differences, can improve data comparability while acknowledging the conflict between maximizing high-quality scanner capabilities and maintaining standardization. Quantitative MRI offers another approach, focusing on absolute unit measurements inherently comparable across sites (Cooper et al., 2020; Smith, Tournier, Calamante, & Connelly, 2015). Improving access to high-quality MRI scanners through resource sharing and targeted funding for technology upgrades ensures broader participation in comprehensive network neuroscience studies.

### Data Sharing

#### Roadblocks.

Data privacy regulations and subsequent regulatory scrutiny present a critical aspect of data analysis and sharing protocols consensus (cf. roadblock 9). Limited capacity and poor integration of picture archiving and communication systems within research environments hinder seamless data exchange. Systems incompatibilities and varying data formats further complicate transfers between clinical and research platforms. The need for comprehensive open access data registers, including detailed clinical information, represents another essential discrepancy in research infrastructure.

#### Enablers.

The development of standardized, scalable data-sharing platforms enhances research integration and facilitates efficient data transfer. The European Health Data Space pilot exemplifies efforts to create frameworks for health data sharing in Europe, addressing interoperability challenges and legal uncertainties.^5^^,^^6^ Promoting interoperability standards and robust IT infrastructures resolves data format compatibility issues, enabling accurate data exchange. Projects like the Brain Imaging Data Structure (BIDS) provide standardized frameworks for data organization and storage, facilitating collaboration (Gorgolewski et al., 2017). The OpenNeuro database demonstrates how standardized formats can enable large-scale data sharing and integration and support multicenter studies, accelerating translational research (Markiewicz et al., 2021). The FAIR (Findability, Accessibility, Interoperability, and Reusability) principles improve machine-based data usage and support scientific data reuse. These principles extend to research software, recognizing it as a digital object requiring similar standardization (Chue Hong et al., 2022; Wilkinson et al., 2016). Online collections of openly available datasets can serve as foundational resources for an open access data registry.^7^

### Computational Resources

#### Roadblocks.

Network analyses demand substantial computational power, especially for dynamic network modeling and multimodal data integration (Sporns, 2018). The complexity of network computations, particularly graph theoretical measures on large-scale brain networks, creates unique implementation challenges for clinical settings. Standard clinical and research environments often lack the necessary hardware for comprehensive analyses, with advanced diagnostics requiring significant computational capabilities. While large research institutions may access cluster computing and national supercomputing facilities, such as Snellius in the Netherlands, these resources remain largely unavailable to clinical environments.

#### Enablers.

Overcoming these challenges, requires scalable computing infrastructure with enhanced processing power and storage solutions. Optimizing preprocessing complexity and coding efficiency ensures responsible resource utilization. Effective translation needs capable algorithms and user-friendly interfaces developed through multidisciplinary collaboration. Cloud-based solutions offer scalable computing and storage resources on demand. Support from governmental or institutional initiatives can ensure that these upgrades and collaborations align with regulatory standards, data privacy laws, and sustainability targets.

### Interdisciplinary Collaboration

#### Roadblocks.

Translating network neuroscience into clinical practice requires seamless collaboration across disciplines, including neuroanatomy, neurosurgery, neurology, psychology, radiology, data science, bioinformatics, and mathematics (Barabási et al., 2023; Bassett & Sporns, 2017). Differences in terminology, methodologies, and priorities create barriers to collaboration and hinder integration into clinical workflows. Software engineers, manufacturers, data scientists, and clinicians must collaborate to develop tools that integrate smoothly into clinical practice.

#### Enablers.

Joint programs and collaborative research environments foster interdisciplinarity. Financial and institutional support for interdisciplinary projects provides incentives for collaboration. Platforms for regular knowledge exchange, including workshops, conferences, and joint publications, promote integration across disciplines. The European Human Brain Project's EBRAINS^8^ and the European Cooperation in Science and Technology funding organization exemplify such initiatives, offering workshops and fellowship programs, although less specified.^9^

### Industry Collaboration and Commercialization

#### Roadblocks.

The translation of advanced TNN methods into widely used, medically certified software is a critical step toward clinical integration (translational phase T4). However, the disconnect between academic research and commercial medical software development often hinders the integration of state-of-the-art TNN tools into existing clinical platforms. The transition from validated research software to clinical application faces a lengthy, often slow and expensive, regulatory approval process. European regulations (Medical Device Regulation, General Data Protection Regulation [GDPR], AI Act) and the U.S. Food and Drug Administration’s framework for Artificial Intelligence/Machine Learning-based Software as Medical Device (SaMD) impose high validation and safety hurdles.^10^^,^^11^ These requirements sometimes lead practitioners to seek workarounds (Beare et al., 2023).

#### Enablers.

Success requires strategic partnerships between academic researchers and medical software companies. Aligning companies' ability to develop profitable solutions with interdisciplinary academic research insights drives innovation. Industry-in-clinic platforms can facilitate these collaborations. Cooperation with regulatory bodies has the potential to establish clear guidance for TNN software as a medical device and streamline certification procedures. Engaging electronic health record (EHR) providers supports seamless integration of TNN results into patient records. Political decision-makers and patient representatives can promote acceptance and adoption of TNN methods in standard clinical care.

### Operational Efficiency, Integration, and Training

#### Roadblocks.

Clinical workflows prioritize patient safety, time-efficiency, and cost-effectiveness potentially limiting seamless integration of advanced methods and cross-disciplinary collaboration. This operational focus creates practical barriers to implementing advanced network analyses, which often require additional time, resources, and expertise beyond standard clinical procedures. Fast-paced clinical environments struggle to accommodate the computational processing times and complex analyses inherent to network approaches.

The education and training gap presents another significant challenge. Network neuroscience's inherent complexity demands understanding of advanced neuroimaging techniques, computational modeling, and data analysis methods. Many clinicians lack the foundational knowledge needed to effectively integrate these sophisticated tools into their clinical workflows.

#### Enablers.

Integrated digital health records and data management systems streamline information sharing between clinical and research teams. Machine learning and artificial intelligence (AI) can help automate and personalize data analysis, though implementation needs careful considerations of regulatory requirements and ethical implications.

Dedicated hospital teams for health data use and access bridge research and clinical practice, facilitating technology integration while ensuring regulatory compliance and clinical workflow alignment. Continuous feedback between researchers and clinicians safeguards tool development matches clinical needs. Multinational infrastructure, such as eBRAIN-health, offers research platforms for brain modeling and simulation across borders.^12^

Training warrants a multifaceted approach. Clinicians need introduction to data security, privacy, and sharing frameworks. Specialized training programs must address the increasing focus on data-centered medicine, potentially leading to new qualifications or specialist titles. Educational resources like Neuromatch Academy and Brainhack^13^ offer intensive courses (Gau et al., 2021; Neuromatch Academy, 2021). Course content is often freely available on the web, YouTube, ^14^^,^^15^ or GitHub, ^16^ providing material for self-study on demand. Funding programs like *Clinician Scientist* in the United Kingdom and Germany facilitate research alongside clinical duties.^17^

### Ethical and Legal Considerations

#### Roadblocks.

Ethical and legal considerations in patient data use have shaped translational research structures, particularly since the GDPR implementation. While Europe's GDPR broadly regulates all personal information, protections in other regions vary; for example, United States regulations split between the Common Rule and the Health Insurance Portability and Accountability Act (White, Blok, & Calhoun, 2022). The complexity of obtaining informed consent stems from the requirement for detailed information about data use, storage, and disclosure within applicable legal frameworks. Data sovereignty issues between patients, researchers, and institutions lack clear definition and face varying interpretations across countries. Ensuring patient privacy demands robust procedures for (pseudo) anonymization and storage, involving significant resources. These critical concerns around patient consent, data ownership, privacy, and ethical implications can delay TNN implementation.

#### Enablers.

Developing these frameworks necessitates simultaneous involvement of ethicists, legal experts, patient representatives, and policymakers. Such collaborative efforts prove essential in the broader context of AI healthcare development (Bouderhem, 2024). Promoting transparency in data use and ensuring robust consent procedures build societal trust and acceptance. The establishment of harmonized national guidelines for clinical research with patient data necessitates a joint effort between the research community, patients, and governmental representatives.

## OUTLOOK

### Bridging Clinical Insight With Network Neuroscience and Technical Advances

Our understanding of cognition and functional capacity as dynamic interactions between brain structure and function is driven by research on functional impairments in neurological conditions like stroke, gliomas, and schizophrenia (Duffau, 2015; Penfield & Rasmussen, 1950; Zhang et al., 2021). Current research reveals how synchronized oscillations in coupled neuronal populations illustrate brain network communication and coordination (Basile et al., 2022; Schirner, Kong, Yeo, Deco, & Ritter, 2022). Studies demonstrate pathological protein spread through communicating neurons and neurotransmitter modulation of brain network dynamics during cognitive tasks, shedding light on mechanisms underlying various brain disorders (Braun et al., 2021; Vogel et al., 2020).

Interindividual variability in network perspectives, particularly in white matter phenotypes and structural connections, holds promise for refining treatment procedures. Preliminary evidence suggests that these differences may influence disease progression and treatment response (Croxson et al., 2018; Forkel, Friedrich, et al., 2022; Jung et al., 2019; Venkatesh et al., 2019). Exploring structural connectivity variations helps assess individual task performance differences, differentiate targeted stimulation effects, and drive personalized therapeutics. These individual differences inform models that can tailor interventions to specific needs, enhancing treatment effectiveness (Bansal, Medaglia, Bassett, Vettel, & Muldoon, 2018).

While big data approaches offer numerous advantages, smaller interventional studies in network neuroscience maintain important value. These studies, particularly in clinical neurology, often provide critical causal insights readily translatable to clinical practice. Investigations using direct cortical stimulation in epilepsy patients, for instance, have revealed invaluable information about the causal role of specific network nodes in cognitive functions (Parvizi, Rangarajan, Shirer, Desai, & Greicius, 2013). Thus, balancing these targeted, mechanistic investigations with larger observational studies is central for advancing translational network neuroscience.

The clinical sector should actively promote TNN beyond individual advanced analyses, for example, by improving patient care documentation. This includes systematic recording of clinical findings, neuropsychological status, and quality of life metrics with greater detail, quality, and standardization. TNN models require development and training with meaningful patient outcomes, necessitating diverse patient representative involvement in outcome definition.

The continuous pursuit of improved image quality and of the advanced method integration remains essential. These advancements in clinical domains drive TNN progress, ultimately enabling more personalized and effective patient care.

### Encouraging Interoperability and Standardization

Encouraging collaboration and data exchange between different centers requires a focus on interoperability and standardization. Integrated data-sharing platforms, consensus on minimal common protocol standards and file formats, like BIDS provide pivotal support (Gorgolewski et al., 2017). Privacy-compliant centralized databases enhance methodology reliability and standardization. A minimum common protocol approach ensures consistency while allowing centers to optimize their resource utilization.

Major initiatives like the Human Connectome Project^18^ and EBRAINS exemplify the impact of standardized acquisitions, methods development, and the insights gained from open access to high-quality datasets. EBRAINS 2.0 targets data harmonization and combining multimodal data, focusing on brain organization, disease mechanisms, and biomarker development^19^ (Amunts et al., 2023; Van Essen et al., 2012). The UK Biobank is another critical infrastructure project, although access has recently been limited by project approval processes, substantial funding requirements, and restricted analysis capabilities within its cloud platform (Sudlow et al., 2015). These infrastructures enable optimized information extraction through open code, improving transparency and reproducibility (Hope et al., 2023; Talozzi et al., 2023; Zalesky et al., 2010).

Clinical cohorts, typically small and variable in data quality, benefit particularly from acquisition standardization and harmonization tools. These approaches facilitate increased statistical power and enable complex model training to identify disease mechanisms and patterns. While training models on heterogeneous data present challenges in noise management and bias control, it can produce more adaptable and robust models (van der Voort et al., 2023). These models better capture real-world variability, improving generalization across diverse populations and enhancing predictive accuracy for personalized treatment. Recent developments of liquid neural networks, which adapt dynamically to changing data streams, show promise in improving model robustness—as demonstrated in adaptive digital twinning (Hasani et al., 2022).

### Enabling Translation

Smooth data exchange in clinical workflows, while essential, remains far from routine (Mäkelä, Vitikainen, Laakso, & Mäkelä, 2015). However, current efforts aim to establish more integrated clinical research environments in digital health (Rieke et al., 2020). The Virtual Research Environment of the Berlin Institute of Health with Charité - Universitaetsmedizin Berlin^20^ exemplifies progress in radiological image data workflows. This environment enables efficient data cataloging, supports various working environments, and ensures interoperability with international data communities, including those within the European Open Science Cloud^21^ framework, such as the Virtual Brain Cloud^22^ and the Human Brain Project.^23^ Such environments enable seamless data migration across platforms while adhering to data governance best practices, including the EU's GDPR compliance. They support data management standards like BIDS and enable analysis, visualization, and clinical application and facilitate federated learning across institutions, enabling more comprehensive use of medical data (Bradshaw et al., 2023; Gorgolewski et al., 2017; Rieke et al., 2020).

### Conclusion

Advances in TNN rely on state-of-the-art neuroimaging and stimulation technologies, leveraging the field's focus on precision and patient-centered medicine (Aerts et al., 2018, 2020; Engelhardt, Grittner, Krieg, & Picht, 2023; Fekonja et al., 2021, 2022; Forkel, Labache, Nachev, Thiebaut de Schotten, & Hesling, 2022; Shams et al., 2022, 2023; Thiebaut de Schotten & Forkel, 2022). While network neuroscience currently faces substantial challenges in establishing robust clinical applications, pivotal studies demonstrated that TNN principles can improve treatment outcomes (Fekonja et al., 2019, 2021; Matthews & Hampshire, 2016; Reisch et al., 2022; Salvalaggio et al., 2023; Schilling et al., 2021; Shams et al., 2022, 2023; Stam, 2014; Tuncer et al., 2021).

Knowledge dissemination and methodological advancement depend critically on consortia and data-sharing initiatives (Thiebaut de Schotten & Forkel, 2022). However, differences in data collection, preprocessing, and analysis methods represent ongoing challenges that need attention (Lioumis & Rosanova, 2022). This methodological heterogeneity, combined with limited clinical validation, highlights key developments needed for reliable clinical translation.

Scientific goals must maintain precise alignment with clinical realities. The field requires considerable developmental work to bridge the gap between research findings and clinical routine applications.

This is our call to action: We must address methodological challenges while fostering interdisciplinary collaboration, standardizing methods, and ensuring strong ethical and legal framework to advance personalized medicine. The translation from promising research tool to validated clinical application requires versatile, interdisciplinary teams capable of integrating diverse expertise. Through systematic validation, methodological refinement, and evidence-based assessment of capabilities, these efforts will ultimately improve patient treatment and lead the field of network neuroscience into a new era of clinical relevance.

## AUTHOR CONTRIBUTIONS

Lucius Samo Fekonja: Conceptualization; Funding acquisition; Project administration; Supervision; Writing – original draft; Writing – review & editing. Stephanie J. Forkel: Conceptualization; Writing – original draft; Writing – review & editing. Dogu Baran Aydogan: Conceptualization; Writing – original draft; Writing – review & editing. Pantelis Lioumis: Conceptualization; Writing – original draft; Writing – review & editing. Alberto Cacciola: Conceptualization; Writing – original draft; Writing – review & editing. Carolin Weiß Lucas: Conceptualization; Writing – original draft; Writing – review & editing. Jacques-Donald Tournier: Conceptualization; Writing – original draft; Writing – review & editing. Francesco Vergani: Conceptualization; Writing – original draft; Writing – review & editing. Petra Ritter: Conceptualization; Writing – original draft; Writing – review & editing. Robert Schenk: Conceptualization; Writing – original draft; Writing – review & editing. Boshra Shams: Writing – original draft; Writing – review & editing. Melina Julia Engelhardt: Conceptualization; Writing – original draft; Writing – review & editing. Thomas Picht: Conceptualization; Funding acquisition; Supervision; Writing – original draft; Writing – review & editing.

## FUNDING INFORMATION

Lucius Samo Fekonja, Deutsche Forschungsgemeinschaft (https://dx.doi.org/10.13039/501100001659), Award ID: EXC 2025—390648296. Lucius Samo Fekonja, Deutsche Forschungsgemeinschaft (https://dx.doi.org/10.13039/501100001659), Award ID: SPP 2041, Project number 455227709. Thomas Picht, Deutsche Forschungsgemeinschaft (https://dx.doi.org/10.13039/501100001659), Award ID: EXC 2025—390648296. Thomas Picht, Deutsche Forschungsgemeinschaft (https://dx.doi.org/10.13039/501100001659), Award ID: SPP 2041, Project number 455227709. Petra Ritter, Deutsche Forschungsgemeinschaft (https://dx.doi.org/10.13039/501100001659), Award ID: SPP 2041, Project number 455227709. Stephanie Forkel, Donders Mohrmann Fellowship No. 2401512 (NEUROVARIABILITY).

## Notes

^1^ Tahedl, M. (2024). B.A.T.M.A.N.: Basic and Advanced Tractography with MRtrix for All Neurophiles. Retrieved from https://doi.org/10.17605/OSF.IO/FKYHT.^2^ Welcome to the MRtrix3 user documentation!—MRtrix3 3.0 documentation. (n.d.). Retrieved from https://mrtrix.readthedocs.io/en/latest/.^3^ Welcome to Andy’s Brain Book!—Andy’s Brain Book 1.0 documentation. (n.d.). Retrieved from https://andysbrainbook.readthedocs.io/en/latest/.^4^ DSI-Studio: A tractography software tool for diffusion MRI analysis. (n.d.). Retrieved from https://dsi-studio.labsolver.org/.^5^ European Health Data Space. (2024, April 24). Retrieved from https://health.ec.europa.eu/ehealth-digital-health-and-care/european-health-data-space_en.^6^ EHDS - EBRAINS. (n.d.). Retrieved from https://www.ebrains.eu/projects/ehds.^7^ OpenDataSets - Methods. (n.d.). Retrieved from https://imaging.mrc-cbu.cam.ac.uk/methods/OpenDatasets.^8^ EBRAINS: Europe’s Research Infrastructure for Brain Research - EBRAINS. (n.d.). Retrieved from https://www.ebrains.eu/.^9^ COST - European Cooperation in Science and Technology. (2024, November 29). COST | European Cooperation in Science and Technology. Retrieved from https://www.cost.eu/.^10^ U.S. Food and Drug Administration. (n.d.). Software as a Medical Device (SaMD). Retrieved from https://www.fda.gov/medical-devices/digital-health-center-excellence/software-medical-device-samd.^11^ AI Act enters into force. (2024, August 1). Retrieved from https://commission.europa.eu/news/ai-act-enters-force-2024-08-01_en#:~:text=The%20Act%20aims%20to%20foster,%2C%20safety%2C%20and%20fundamental%20rights.^12^ eBRAIN-Health. (n.d.). Retrieved from https://ebrain-health.eu/home.html.^13^ Brainhack. (n.d.). Retrieved from https://brainhack.org/.^14^ Clinical Neuroanatomy Seminars. (n.d.). Home [YouTube Channel]. Retrieved from https://www.youtube.com/c/ClinicalNeuroanatomySeminars.^15^ Organization for Human Brain Mapping [OHBM]. (n.d.). Home [YouTube Channel]. Retrieved from https://www.youtube.com/@OHBM.^16^ Neuromatch Academy (NMA). (2021). Retrieved from https://github.com/NeuromatchAcademy.^17^ Clinician Scientist programmes of the DFG. (n.d.). Retrieved from https://www.dfg.de/en/research-funding/funding-initiative/clinician-scientist-programme.^18^ The Human Connectome Projects. (n.d.). Human Connectome Project - Homepage. Retrieved from https://www.humanconnectome.org/.^19^ EBRAINS research infrastructure secures €38 million in funding for new phase of digital neuroscience - EBRAINS. (n.d.). Retrieved from https://www.ebrains.eu/news-and-events/ebrains-research-infrastructure-secures-38-million-in-funding-for-new-phase-of-digital-neuroscience.^20^ Berliner Institut für Gesundheitsforschung - Charité und Max-Delbrück-Centrum. (2021, July 29). BIH/Charité Virtual Research Environment - BIH at Charité. Retrieved from https://www.bihealth.org/en/translation/network/digital-medicine/bihcharite-virtual-research-environment.^21^ Research and innovation. (n.d.). Retrieved from https://commission.europa.eu/research-and-innovation_en?pg=open-science-cloud.^22^ TVB-Cloud. (n.d.). Retrieved from https://virtualbraincloud-2020.eu/tvb-cloud-main.html.^23^ Human Brain Project. (n.d.). Retrieved from https://www.humanbrainproject.eu/en/.

## TECHNICAL TERMS

Network neuroscience: The study of the organization and function of the brain using network theory to analyze neuronal connections and interactions.
Structural connectivity: Modeled white matter pathways between brain regions, derived from diffusion MRI techniques.
Functional connectivity: Statistical relationships between brain regions, reflecting the synchronization of neuronal activity as measured by fMRI.
Effective connectivity: Causal effects of one brain region on another, modeled to infer the direction and strength of neural interactions.
Connectomics: The field of mapping comprehensive neuronal connections (the connectome) to study brain network organization through structural, functional, and effective connectivity.
Translational network neuroscience: The application of network neuroscience methods to clinical practice, bridging research and clinical practice.
Graph-theoretical measures: Mathematical metrics that quantify brain network properties at nodal (local) and network (global) levels, including segregation, integration, and dynamics.
Personalized medicine: An approach to healthcare that uses individual characteristics to tailor diagnostics and treatments to specific patient needs.
Dynamic functional connectivity: Time-varying functional connectivity patterns showing the reorganization of brain networks during different tasks or cognitive states.
Translational medicine: The process of bridging research and clinical practice to improve medical diagnosis, treatments, and patient outcomes.
